# Efficient and Accurate 3D Thickness Measurement in Vessel Wall Imaging: Overcoming Limitations of 2D Approaches Using the Laplacian Method

**DOI:** 10.3390/jcdd11080249

**Published:** 2024-08-15

**Authors:** SeyyedKazem HashemizadehKolowri, Ebru Yaman Akcicek, Halit Akcicek, Xiaodong Ma, Marina S. Ferguson, Niranjan Balu, Thomas S. Hatsukami, Chun Yuan

**Affiliations:** 1 Department of Radiology and Imaging Sciences, University of Utah, Salt Lake City, UT 84108, USA; ebru.akcicek@utah.edu (E.Y.A.); halit.akcicek@utah.edu (H.A.); xiaodong.ma@hsc.utah.edu (X.M.); chun.yuan@hsc.utah.edu (C.Y.); 2 Department of Radiology, University of Washington, Seattle, WA 98195, USA; msf2@uw.edu (M.S.F.); ninja@uw.edu (N.B.); 3 Department of Surgery, Division of Vascular Surgery, University of Washington, Seattle, WA 98195, USA; tomhat@uw.edu

**Keywords:** vessel wall imaging, vessel wall 3D thickness measurement, Laplace’s equation, convolutions

## Abstract

The clinical significance of measuring vessel wall thickness is widely acknowledged. Recent advancements have enabled high-resolution 3D scans of arteries and precise segmentation of their lumens and outer walls; however, most existing methods for assessing vessel wall thickness are 2D. Despite being valuable, reproducibility and accuracy of 2D techniques depend on the extracted 2D slices. Additionally, these methods fail to fully account for variations in wall thickness in all dimensions. Furthermore, most existing approaches are difficult to be extended into 3D and their measurements lack spatial localization and are primarily confined to lumen boundaries. We advocate for a shift in perspective towards recognizing vessel wall thickness measurement as inherently a 3D challenge and propose adapting the Laplacian method as an outstanding alternative. The Laplacian method is implemented using convolutions, ensuring its efficient and rapid execution on deep learning platforms. Experiments using digital phantoms and vessel wall imaging data are conducted to showcase the accuracy, reproducibility, and localization capabilities of the proposed approach. The proposed method produce consistent outcomes that remain independent of centerlines and 2D slices. Notably, this approach is applicable in both 2D and 3D scenarios. It allows for voxel-wise quantification of wall thickness, enabling precise identification of wall volumes exhibiting abnormal wall thickness. Our research highlights the urgency of transitioning to 3D methodologies for vessel wall thickness measurement. Such a transition not only acknowledges the intricate spatial variations of vessel walls, but also opens doors to more accurate, localized, and insightful diagnostic insights.

## 1. Introduction

Cardiovascular disease remains a prominent cause of morbidity and mortality worldwide, necessitating the development of precise diagnostic and preventive strategies. The connection between vessel wall thickness and cardiovascular outcomes has been extensively established [1,2,3,4,5,6,7,8,9], underscoring the clinical importance of accurately measuring and characterizing arterial wall thickness. While various imaging techniques have been developed for this purpose, the fundamentally three-dimensional (3D) nature of vessel wall thickness measurement has often been overlooked in existing approaches.

B-mode ultrasound, a widely employed method [1,5,10,11], offers real-time imaging and non-invasiveness, allowing accurate vessel wall thickness measurement at relatively low cost. Magnetic resonance imaging (MRI), another modality for vessel wall imaging, provides exceptional soft tissue contrast and spatial resolution, yielding precise vessel wall thickness measurements with high reproducibility [12,13,14]. Recent advancement in imaging technologies has led to the emergence of various techniques for imaging vessel walls and characterizing atherosclerotic lesions, potentially improving risk assessment and patient management [15,16,17,18]. Additionally, recent progress in deep learning methodologies, has improved lumen and outer wall segmentation accuracy [19,20,21], highlighting the potential for enhanced vessel wall thickness measurement methodologies.

**Figure 1:**
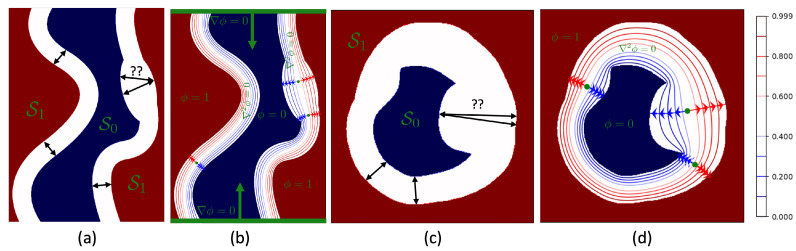
(**a**,**c**) Thickness measurement for parallel (double arrows) versus non-parallel (double arrows with ??) boundaries. (**b**,**d**) Subdividing the space between the two boundaries into smaller regions using parallel sublayers. By summing up the distances between these parallel sublayers, the thickness can be computed. The Laplace’s Equation ($\nabla^{2}\phi =0$), and Dirichlet (${\phi |}_{S_{0}}{=0.00,\phi |}_{S_{1}}=1.00$) and Neumann (${\nabla \phi |}_{caps}=\vec{0}$) boundary conditions are shown. At any given point, the thickness is determined by the summation of the lengths of two paths: a positive path (illustrated in red) extending from the specified point to the boundary with the higher potential, and a negative path (depicted in blue) extending from the specified point to the boundary with the lower potential.

Traditionally, vessel wall thickness measurement has relied on two-dimensional (2D) approaches [14,22,23,24,25,26,27], which involve extracting 2D slices from 3D volumetric data. While these techniques allow calculation of standardized parameters, such as mean/maximum wall thickness and the normalized wall index, they inherently neglect the complete 3D nature of vessel walls [28]. Overlooking wall thickness variations in all three spatial directions, limits capacity of these methods to capture the holistic 3D characteristics of vessel walls, leading to inherent limitations that hinder the full clinical potential of vessel wall imaging techniques. A primary limitation of existing approaches is their reliance on the extracted 2D slices. In the case of straight vessels, the thickness measurement using cross-sectional 2D slices is reasonable; however, twisted arteries require extraction of cross-sectional slices that are perpendicular to luminal centerlines. Even with perfect centerline calculation, it is not always possible to extract cross-sectional slices perpendicular to vessel walls at all locations in highly tortuous vessels (see Figure 1a). Additionally, the lumen and outer wall boundaries in the extracted cross-sectional slices might be very complex (see Figure 1c), which makes wall thickness measurement very challenging even only in two dimensions. Furthermore, existing methods often only measure wall thickness at the lumen boundary, disregarding vital information about thickness variations across the entire vessel wall volume. As a result, comprehensive evaluations of wall thickness distribution throughout the entire wall volume remain elusive using conventional 2D techniques.

To address these limitations and advance vessel wall thickness assessment, this study proposes adopting the Laplacian method [29] as an alternative measurement technique. By relying on the 3D geometric properties of vessel walls, the Laplacian method offers accurate and reproducible measurements while providing crucial localization insights into regions exhibiting abnormal wall thickening. Notably, this approach can be efficiently and expeditiously executed on deep learning (DL) platforms utilizing graphics processing units (GPUs), facilitating its integration into clinical workflows. The Laplacian method introduced in this study confers several advantages over existing approaches. First, it uses all available spatial dimensions in measuring the wall thickness. Second, its applicability spans both 2D and 3D scenarios, ensuring seamless transition between imaging modalities and offering adaptability in clinical settings. Third, by enabling voxel-wise measurements of arterial wall thickness, the Laplacian method captures thickness variations not only at lumen boundaries, but at the entire vessel wall volume. This allows for the 3D identification of regions with abnormal wall thickness, enabling precise localization of pathological changes and refining risk stratification.

In the subsequent sections, we will detail the Laplacian method’s implementation through convolutions, demonstrating its efficient execution on DL platforms with GPU acceleration. Additionally, we will present experimental results showcasing the accuracy, reproducibility, and localization capabilities of this proposed approach, emphasizing its potential for enhanced cardiovascular risk diagnosis and prevention.

## 2. Theory

Thickness is a commonly employed concept that describes the distance separating two boundaries of an object or material. Typically, when dealing with 2D and 3D objects, their boundaries are specified using 1D curves and 2D surfaces, respectively. Consequently, the measurement of thickness becomes equivalent to measuring the distance between two 1D curves or two 2D surfaces. In cases where the boundaries are parallel, determining the distance (and thus, the thickness) is straightforward, as the distance between the two boundaries remains unchanged at all locations. However, when dealing with non-parallel boundaries, establishing a generalized and self-consistent definition of thickness becomes less straightforward, as depicted in Figure 1a,c.

Fortunately, for most practical applications, including the measurement of vessel wall thickness, the space between the two boundaries of interest can be subdivided into smaller regions using numerous infinitesimally proximate sublayers, all of which are parallel, as illustrated in Figure 1b,d. By summing up the distances between these parallel sublayers, the overall distance between the two original boundaries can be computed. To establish a mathematical formalization of this process, we denote the two boundaries by $S_{0}$ and $S_{1}$. To identify the parallel sublayers, we assign a scalar or a potential field ϕ to the space between $S_{0}$ and $S_{1}$, and assign different values (e.g., ${\phi |}_{S_{0}}=0.00$ and ${\phi |}_{S_{1}}=1.00$) to the boundaries to distinguish between them. By imposing a constraint on the divergence of the potential field, such that $\nabla^{2}\phi =0$ within the space between the two boundaries, the resulting contours or equipotential surfaces of the potential field do not diverge at any point, thereby forming parallel sublayers. A brief overview of the Laplacian operator and its application is presented in the Supporting Information. Consequently, the problem of thickness measurement reduces to solving the following partial differential equation:

(1) $$\nabla^{2}\phi =0\;s.t.\;\phi {{|}_{S_{0}}=0.00,\;\phi |}_{S_{1}}=1.00.$$

The above equation and boundary conditions are referred to as Laplace’s equation and Dirichlet conditions, respectively. Laplace’s equation is encountered in a wide array of mathematical and physical problems. Another type of boundary condition commonly associated with Laplace’s equation is known as the Neumann boundary condition, which imposes restrictions on the gradients of the potential field. In the context of measuring vessel wall thickness, the Neumann conditions, i.e., ${\nabla \phi |}_{caps}=\vec{0}$, can be applied to the end caps of the vessels, assuming that the vessels are extend uniformly when measuring the thickness at the end caps. After finding the solution to Equation (1), the normalized gradient vector field in the space between two boundaries can be obtained using $\vec{n}=\nabla \phi /∥\nabla \phi ∥$. Given the normalized gradient vector field, the thickness at a specific location, $\vec{P}$, can be determined by adding lengths of two distinct paths, as visually depicted in Figure 1b,d. One path is characterized by a positive trajectory originating from $\vec{P}$ and extending towards the boundary with the higher potential. The other path represents a negative trajectory proceeding from $\vec{P}$ and terminating at the boundary with the lower potential. Notably, both paths are oriented perpendicularly to the potential field. The procedure for this thickness measurement can be realized following the steps outlined in Algorithm 1. In this algorithm, *T* is the measured thickness at point $\vec{P}$, $T^{+}$ is the length of the positive path which is equal to the distance to the outer wall, $T^{-}$ is the length of the negative path which is equal to the distance to the lumen, and γ is the step size which is set to be smaller than a voxel size, e.g., h/4.
**Algorithm 1:** Computing the thickness
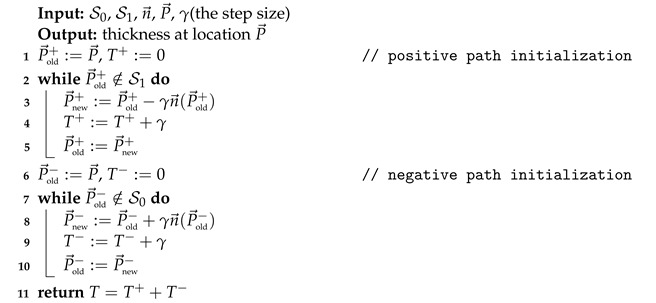


In order to solve partial differential equations such as the Laplace’s equation stated in Equation (1), numerical methods are commonly utilized. These techniques predominantly involve estimating partial derivatives of the potential field by employing finite difference approximations on either regular or non-regular grids. In the context of medical images, the boundaries of pixels/voxels within the image can be employed as a regular Cartesian grid for the purpose of finite difference approximation. In the case of a two-dimensional image, the Laplacian operator applied to the potential field, denoted by $\nabla^{2}\phi$, can be approximated using the five-point stencil finite difference formula [30], which is expressed as follows:

(2) $$\nabla^{2}\phi \left(x,y\right)=\frac{1}{h^{2}}\left[\phi \left(x-h,y\right)+\phi \left(x+h,y\right)+\phi \left(x,y+h\right)+\phi \left(x,y-h\right)-4\phi \left(x,y\right)\right]$$

where *h* represents the grid spacing. For image processing applications, we assign h=1 to reflect a one-pixel spacing. Consequently, Equation (2) can be reformulated using a simpler notation as follows:

(3) $$\nabla^{2}\phi_{0,0}=\phi_{-1,0}+\phi_{1,0}+\phi_{0,1}+\phi_{0,-1}-4\phi_{0,0}.$$

To obtain a numerical solution for Laplace’s equation, as stated in Equation (1), it is possible to set the right-hand side of Equation (3) to zero. By iteratively enforcing this condition along with the boundary conditions, convergence towards a solution can be achieved. To be more precise, the potential field in the *i*-th iteration can be updated using the following recursive expression:

(4) $$\phi_{0,0}^{\left(i+1\right)}=\frac{1}{4}\left[\phi_{-1,0}^{\left(i\right)}+\phi_{1,0}^{\left(i\right)}+\phi_{0,1}^{\left(i\right)}+\phi_{0,-1}^{\left(i\right)}\right].$$

The above equation demonstrates the updating process of an individual pixel. To apply this process to the entire potential field, denoted by ϕ and represented as a 2D image, a 2D convolution operation can be utilized in the following manner:

(5) $$\phi^{\left(i+1\right)}=\frac{1}{4}\left[\begin{array}{ccc} 0 & 1 & 0\\ 1 & 0 & 1\\ 0 & 1 & 0 \end{array}\right]⊛\phi^{\left(i\right)}=K_{5-\mathrm{pt}}⊛\phi^{\left(i\right)}$$

We note that modern deep learning platforms facilitate rapid and efficient computation of convolutions by leveraging GPU resources. Consequently, Equation (5) enables the implementation of thickness measurement in an efficient manner. To start solving Equation (5), the potential field can be initialized as the average value between the two potential values at the boundaries. Moreover, the attainment of convergence can be evaluated by examining the change in total energy of the potential field, as expressed by the following equation:

(6) $$E^{\left(i+1\right)}=∥\phi^{\left(i+1\right)}-\phi^{\left(i\right)}∥$$

By consolidating all the aforementioned information, the solution to Laplace’s equation can be derived by following the procedural steps outlined in Algorithm 2.
**Algorithm 2:** Solving the Laplace’s equation using convolutions
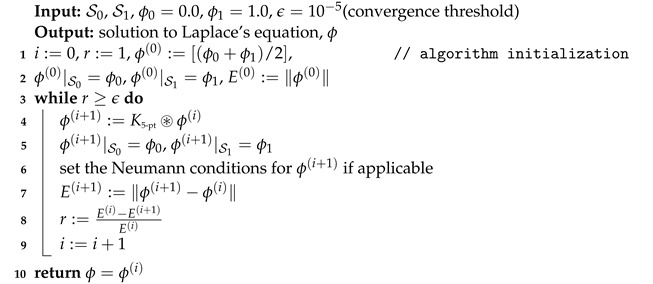


We note that for thickness measurement, as demonstrated in Algorithm 1, the potential field’s gradient is needed. The computation of the gradient of the potential field can be accomplished efficiently by employing the Sobel–Feldman operator [31] and implementing it using convolutions, as follows:

(7a) $$\phi_{x}=\frac{1}{8}\left[\begin{array}{ccc} 1 & 0 & -1\\ 2 & 0 & -2\\ 1 & 0 & -1 \end{array}\right]⊛\phi =G_{x}⊛\phi$$

(7b) $$\phi_{y}=\frac{1}{8}\left[\begin{array}{ccc} 1 & 2 & 1\\ 0 & 0 & 0\\ -1 & -2 & -1 \end{array}\right]⊛\phi =G_{y}⊛\phi$$

The extension of the aforementioned algorithms for 3D thickness measurement is straightforward and accomplished by substituting 2D operations with their corresponding 3D counterparts as discussed in Appendix A. Furthermore, enhancing the robustness of the Laplacian and gradient operators can be achieved by employing alternative kernels in the convolutions. These modifications are elaborated upon in greater detail in Appendix B.

## 3. Materials and Methods

In this section, we present a series of experiments crafted to assess the accuracy and reproducibility of the proposed Laplacian method for measuring vessel wall thickness. By conducting a variety of experiments that encompass both 2D and 3D scenarios, our aim is to evaluate the effectiveness of the Laplacian method across different imaging modalities and clinical situations.

### 3.1. Thickness Measurement of 2D Digital Phantoms

In the first experiment, digital phantoms with circular and elliptical ring geometries were employed to simulate vessel wall thickness measurements in 2D scenarios. The circular ring had an inner radius of r=1.5mm with a uniform thickness of d=1.5mm. The elliptical ring, showcasing a more complex geometry, had a major axis of $r_{1}=1.5\;\mathrm{mm}$ and a minor axis of $r_{2}=1\;\mathrm{mm}$ with a uniform thickness of d=1.5mm. Thickness measurements using the Laplacian method were conducted at isotropic spatial resolutions of 2, 5, 10, 20, 50, 100, and 200 pixels/mm, respectively.

### 3.2. Outer Wall Thickness Measurement in 2D Histology Images

In the second experiment, we utilized histology images from two carotid artery samples. After obtaining informed consent, specimens from carotid endarterectomy were acquired. The specimens underwent formalin fixation, formic acid decalcification, and paraffin embedding. Serial Sections (10 μm thick) were made at specific intervals in the common carotid artery (CCA) and internal carotid artery (ICA) and stained with hematoxylin and eosin (H&E). An experienced research scientist, performed manual segmentation on 2D histology images, based on histopathologic classification, particularly following Stary et al.’s criteria [32], to identify outer wall and plaque compositional features.

Regions of interest, including lipid pools, necrotic cores, recent and late hemorrhages, calcifications, and loose extracellular matrices, were defined according to established criteria outlined by various authors [33,34,35,36]. Lipid pools, representing early lesions with distinct spaces composed of phospholipids and cholesterol, are visualized as acellular spaces through H&E. Necrotic cores, characterized by lipid and necrotic debris, may contain recent or late hemorrhage. Recent hemorrhages exhibit a mixture of intact and degenerating red cells, while late hemorrhages present eosinophilic material with cholesterol clefts. Calcifications show hydroxyapatite crystals, and loose extracellular matrices exhibit loosely organized fibers with smooth muscle cells and micro-vessels. These defined regions provided insights into carotid artery pathology.

The vessel wall thickness measurements using the Laplacian method were conducted on digital histology images with spatial resolutions of 100 and 180 pixel/mm, respectively. For each region of interest (ROI), the area, the normalized index, the mean and standard deviation of the wall thickness, as well as the mean and standard deviation of the distance to the lumen, were computed. The normalized index is defined as the area of ROI divided by the total vessel area. We note that the wall thickness and distance to lumen measurements are obtained using the Laplacian method, while the lumen area and the normalized wall index are computed directly from the manually drawn segmentation masks. On a CentOS server equipped with Intel^®^ Xeon^®^ E5-2620 v4 @2.10 GHz CPUs and two Nvidia^®^ Tesla P40 GPUs, each with 24 GB of memory, the thickness measurement algorithm took 126 and 307 s to run for two different samples, respectively.

### 3.3. Thickness Measurements of 3D Digital Phantoms

In the third experiment, we utilized three digital phantoms, including a half-spherical shell, a half-ellipsoid shell, and a bent pipe, to simulate thickness measurements of 3D objects. The half-spherical shell had an inner radius of r=1.5mm. The half-ellipsoid shell had a major axis of $r_{1}=2\;\mathrm{mm}$ and minor axes of $r_{2}=1.5$ and $r_{3}=1\;\mathrm{mm}$. The bent pipe had an inner radius of r=1.5mm and a quarter-circle axis with the radius of R=4.5mm. All three phantoms had a uniform thickness of d=1.5mm. The thickness measurements using the Laplacian method were performed for isotropic spatial resolutions of 2, 5, and 10 voxel/mm, respectively. We note that thickness measurements at higher spatial resolutions are not practically feasible because of huge computational requirements.

### 3.4. 3D Vessel Wall Thickness Measurement of Carotid Arteries in Black Blood MR Images

In the final experiment, we evaluated carotid vessel wall thickness measurements in MR images acquired using a 3D black blood contrast sequence. A healthy female volunteer aged 25 years, after providing informed consent and receiving institutional review board (IRB) approval, underwent scanning twice on a Siemens 3T MRI scanner (Prisma-fit, Erlangen, Germany). The follow-up scan took place one month after the baseline scan. For both scans, we utilized a custom-designed neck-shape-specific carotid coil (7 channels) [37], combined with a Siemens 20-channel head/neck coil. The black blood contrast was achieved using the 3D motion-sensitized driven equilibrium (MSDE), prepared rapid gradient echo (3D-MERGE) sequence [15], with the following parameters: field of view (FOV) = 250×160×42 mm^3^, acquistion voxel size = 0.82 × 0.82 × 0.82 mm^3^, interpolated voxel size = 0.41 × 0.41 × 0.41 mm^3^, TE/TR = 4.3/9.1 ms, preparation time = 22 ms, number of segments = 22, number of averages = 2, and scan time = 4 min and 35 s.

The 3D segmentation masks for the lumen and outer wall of the left and right carotid arteries were derived through processing of 3D MERGE imaging data using an in-house artificial intelligence (AI) framework [38,39]. This framework adopts a three-step methodology to process data from black blood vessel wall imaging data. Initially, it detects the locations of the right and left bifurcation slices. Subsequently, these identified slices are utilized to extract axial slices that encompass the bifurcation region, as well as the distal and proximal segments of the carotid arteries. In the next step, precise localization of the carotid arteries is achieved through 3D segmentation of their lumens by employing a 3D U-Net. Finally, the outcomes of this localization process are employed to extract 2D patches along the lumens of both the left and right carotid arteries, which are fed to 2D convolutional neural networks for the final lumen and outer wall segmentation. The segmentation outcomes were reviewed and manually corrected by trained reviewers using the ITK-SNAP 4.X software (Available online: http://www.itksnap.org/, accessed on 10 August 2024) [40], to ensure accuracy. Minor adjustments were made, if necessary, to refine the boundaries of the lumen and outer wall, improving overall segmentation quality.

To enhance thickness measurement accuracy, the segmentation masks were upsampled by a factor of 3 and smoothed using a 3D Gaussian filter before thickness measurement. Vessel wall thickness measurements utilizing the Laplacian method were subsequently performed. Using the same server mentioned in Section 3.2, the 3D thickness measurement algorithm took 227 and 212 s to run for the baseline and follow-up scans, respectively. For comparative analysis of thickness measurement results between the baseline and follow-up scans, the centerline and bifurcation coordinate system for the left and right carotid arteries were obtained using their lumen surfaces according to [41,42]. Lumen surfaces were extracted from the corresponding upsampled lumen masks using the marching cube algorithm. The centerline extraction and bifurcation coordinate system derivation methods have been implemented in an open-source Python package known as the vascular modeling toolkit (VMTK) (Available online: http://www.vmtk.org/, accessed on 10 August 2024) [43]. These routines enable robust extraction of the centerlines and associated metrics, including the centerline length parameter (abscissa) and the radius of the maximum inscribed sphere on the luminal surface. The bifurcation coordinate system establishes a non-uniform rectangular grid on the luminal surface of each branch within the carotid artery. The determination of coordinates involves partitioning the surface in the angular direction into sectors and in the longitudinal direction (along the centerline) into slabs.

Thickness measurement results were compared in two ways. In the first approach, vessel wall thickness was measured on the luminal surface and then averaged over rectangular patches forming the bifurcation coordinate system. In the second approach, vessel wall thickness was measured in all voxels within the outer wall volume, then averaged within a moving cylinder centered at each point along the centerline. The moving cylinder’s axis coincided with the centerline, and its length was equal to the diameter of the maximum inscribed sphere to the luminal surface.

## 4. Results

### 4.1. Thickness Measurement of 2D Digital Phantoms

**Figure 2:**
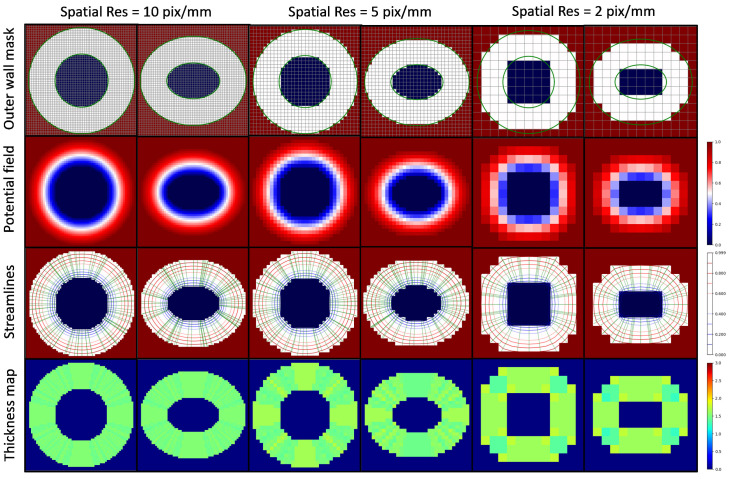
(First row) 2D digital phantoms with circular and elliptical ring geometries. (Second row) Solutions to the Laplace’s Equation. (Third row) Subsets of derived streamlines. (Fourth row) Thickness maps.

**Figure 3:**
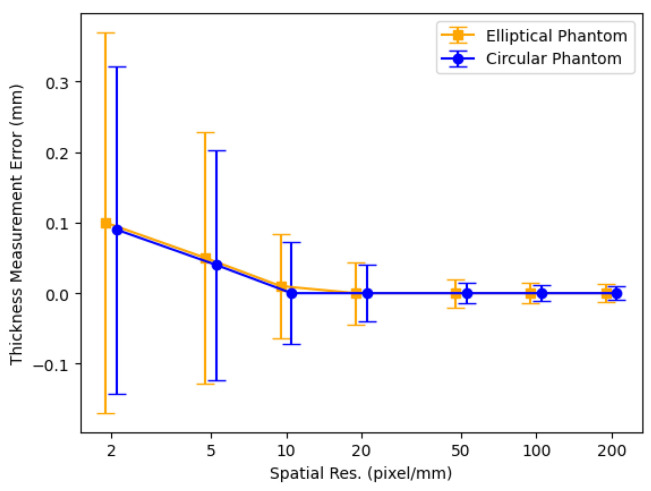
Thickness measurement errors for 2D digital phantoms as spatial resolution is increased.

The results of the first experiment are shown in Figure 2 and Figure 3. Figure 2 depicts circular and elliptical ring geometries (first row), solutions to the Laplace’s Equation (second row), subsets of derived streamlines (third row), and the thickness maps (fourth row) corresponding to spatial resolutions of 2, 5, and 10 pixel/mm. We note that the second row displays a continuous potential field that increases from 0 to 1 as we move from the lumen to the outer wall, representing the solution to Laplace’s equation obtained using Algorithm 2. The third row shows the trajectories of streamlines obtained using Algorithm 1. It is evident from Figure 2 that thickness maps exhibit a nearly uniform distribution around 1.5 mm, thereby affirming the uniform thickness of digital phantoms. Moreover, as the spatial resolution increases, thickness maps display enhanced uniformity, converging toward the 1.5 mm mark. This observation suggests that, at sufficiently high spatial resolutions, the Laplacian method yields accurate thickness measurements.

Figure 3 displays thickness measurement errors for 2D digital phantoms as functions of spatial resolution. The plot reveals that, for higher spatial resolutions, thickness measurement biases approach zero, and the errors remain minimal. Conversely, as spatial resolution decreases, the errors exhibit a proportional increase with pixel size. Additionally, non-zero biases emerge, attributed to pixelation effects that prevent a precise realization of the ring geometry at lower spatial resolutions. Notably, the elliptical phantom manifests marginally higher errors compared to the circular phantom, implying that a more complex geometry tends to yield a larger thickness error compared to a simpler geometry.

### 4.2. Outer Wall Thickness Measurement in 2D Histology Images

**Figure 4:**
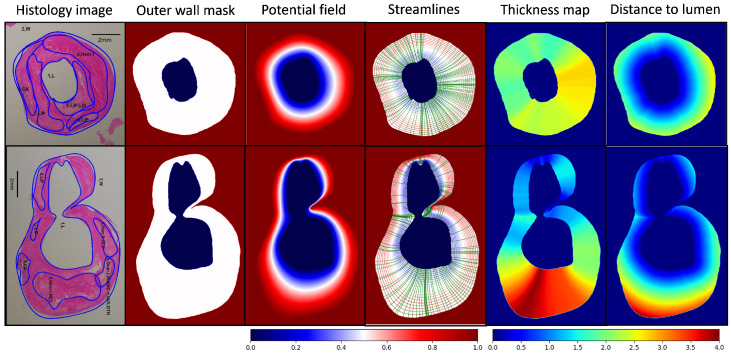
(First column) Histology images of two carotid samples and manual annotation of ROIs. (Second column) Vessel wall segmentation masks. (Third column) Solutions to the Laplace’s Equation. (Fourth column) Contours of potential fields and subsets of corresponding streamlines. (Fifth column) Vessel wall thickness maps. (Sixth column) Distance-to-lumen maps.

The results from the second experiments are presented in Figure 4 and Table 1. In Figure 4, histology images of two carotid samples and manual annotation of ROIs (first column), vessel wall segmentation masks (second column), solutions to the Laplace’s Equation (third column), contours of potential fields and subsets of corresponding streamlines (fourth column), the vessel wall thickness maps (fifth column), and the maps of distance to lumen (sixth column) are shown. We note that the Laplacian method, in addition to thickness measurement, allows for measuring the distance to lumen (length of the negative path in Algorithm 1) and the distance to outer wall (length of the positive path in Algorithm 1). We also see that the Laplacian method is capable of accurately measuring wall thickness for both regular, e.g., sample#1, and complex, e.g., sample#2, vessel wall morphologies. From thickness maps, it is evident that vessel wall regions with hemorrhage exhibit a notably increased wall thickness compared to other regions, which is consistent with the findings reported in previous studies. Additionally, we see that pathologies such as lipid pools are present in wall regions with both normal and abnormal thickness; however, most plaque features are observed in areas with abnormal wall thickening. This implies that diseased vessel walls may not be completely identifiable solely based on the wall thickness measurement; nevertheless, a strong correlation exists between the wall thickness and the presence of plaque components. Looking at distance-to-lumen maps, we see that plaque features are observed both in close proximity to the lumen and away from it, but distances of most plaque components to the lumen appear to be larger than the 1 mm threshold. This observation suggests that wall thickening attributed to plaque development predominantly occurs in areas closer to the outer wall boundary in these two histology samples.

In Table 1, quantitative morphological measurements derived from histology images of carotid samples are presented. The two specimens manifest abnormal wall thickness, as evidenced by the overall mean wall thickness exceeding 2 mm. Additionally, the mean wall thickness within all ROIs corresponding to plaque components, excluding lipid pools, surpasses the threshold of normal wall thickness (1.5 mm). This affirmation aligns with our earlier observation in Figure 4, i.e., that a strong correlation exists between the vessel wall thickening and the presence of plaque. For both samples, ROIs featuring hemorrhage exhibit a relatively large mean wall thickness. Moreover, for all plaque components except lipid pools, the average distance-to-lumen exceeds 1 mm, corroborating the findings observed in Figure 4.

### 4.3. Thickness Measurements of 3D Digital Phantoms

**Figure 5:**
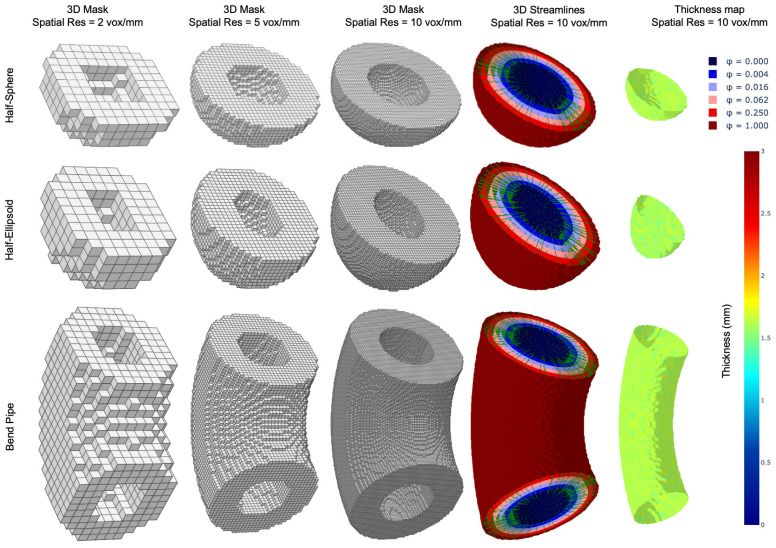
(First–third columns) 3D masks (volumes) of three digital phantoms with isotropic spatial resolutions of 2, 5, and 10 voxel/mm, respectively. (Fourth column) Equipotential surfaces of the potential field (solutions to the Laplace’s equation) and subsets of corresponding orthogonal streamlines for a spatial resolution of 10 voxel/mm. (Fifth column) Thickness maps on the inner surfaces of the digital phantoms for a spatial resolution of 10 voxel/mm.

**Figure 6:**
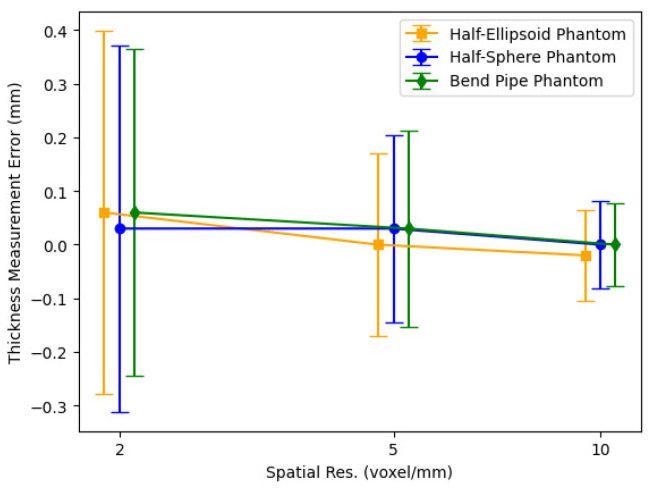
Thickness measurement errors for 3D digital phantoms as spatial resolution is increased.

The outcomes of the third experiment are presented in Figure 5 and Figure 6. Figure 5 illustrates 3D volumes of three digital phantoms with isotropic spatial resolutions of 2, 5, and 10 voxel/mm in the first through third columns, respectively. Additionally, it displays equipotential surfaces of the potential fields, which are solutions to the Laplace’s equation, and subsets of orthogonal streamlines for the spatial resolution of 10 voxel/mm in the fourth column. The fifth column shows thickness maps on the inner surfaces of phantoms for the spatial resolution of 10 voxel/mm. These results indicate that the Laplacian method, employed for measuring thickness in 3D objects, performs comparably well to its application in 2D scenarios. In particular, the fifth column in Figure 5 shows that thickness maps for all three phantoms have a nearly uniform distribution around a value of 1.5 mm. This observation affirms the uniform thickness of digital phantoms, and hence, implies that at a sufficiently high spatial resolution, the Laplacian method provides accurate 3D thickness measurements.

Figure 6 displays the 3D thickness measurement errors for the three digital phantoms, ploted as functions of spatial resolution. The plot indicates that, for the examined spatial resolutions, thickness measurement biases are nearly zero. Furthermore, as the spatial resolution is decreased, the errors demonstrate a proportional increase in accordance with voxel size. A comparison with the thickness measurement errors for 2D phantoms in Figure 3 reveals that biases are smaller in 3D scenarios. This might be attributed to less severe pixelation effects in three dimensions due to increased spatial degree of freedom. Conversely, the measurement errors for 3D phantoms are larger than those of 2D phantoms, as an additional error term from the third dimension contributes to the overall thickness measurement error. We note that all three phantoms exhibit similar thickness measurement errors, suggesting the 3D geometrical complexity of these phantoms are comparable.

### 4.4. 3D Vessel Wall Thickness Measurement of Carotid Arteries in Black Blood MR Images

**Figure 7:**
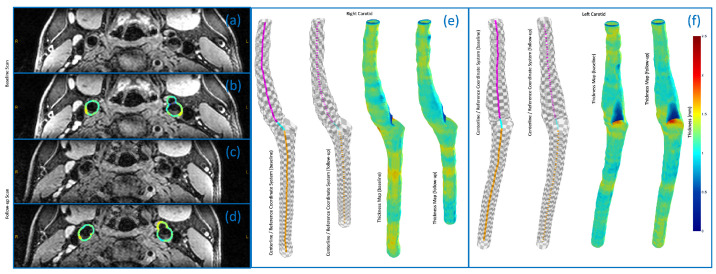
(**a**,**c**) Axial views of 3D MERGE MR imaging data for the baseline and follow-up scans. (**b**,**d**) The corresponding vessel wall thickness maps superimposed on the axial views. (**e**,**f**) Extracted centerlines, bifurcation coordinate systems, and vessel wall thickness maps on the luminal surfaces in baseline and follow-up scans for the right (**e**) and left (**f**) carotid arteries.

**Figure 8:**
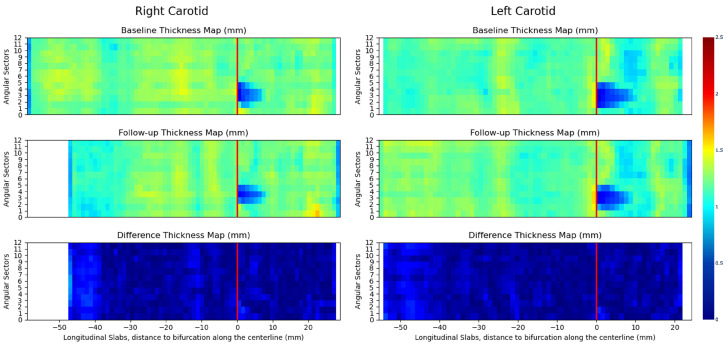
3D vessel wall thickness measurements on luminal surfaces of the left and right carotid arteries in the baseline and follow-up scans. These regular 2D grids represent rectangular patches (derived from bifurcation coordinate system) on the luminal surfaces in an unwrapped and flattened format. Vertical red lines separate 2D grids associated with CCA and ICA branches. Each thickness value on the flattened grid is computed by averaging thickness measurements within the corresponding patch on the luminal surface.

**Figure 9:**
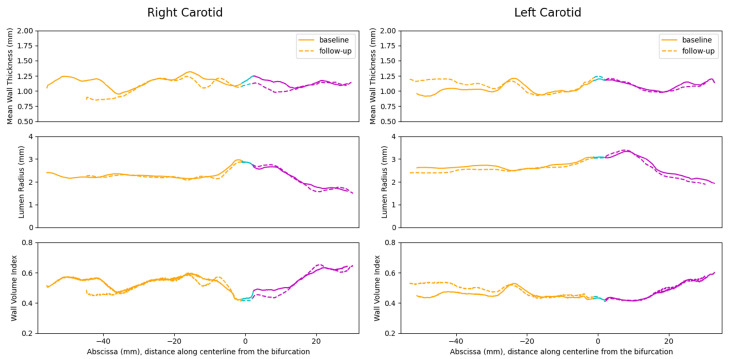
Average 3D vessel wall thickness measurement, luminal radius, and the normalized wall volume index of the left and right carotid arteries along their centerlines for the baseline and follow-up scans. The solid-line and dash-line curves correspond to the baseline and follow-up scans, respectively. Carotid artery branches are color-coded, with orange indicating the CCA, magenta indicating the ICA, and cyan indicating the bifurcation region.

The results from the final experiment are presented in Figure 7, Figure 8 and Figure 9. In the left panel of Figure 7, axial views of 3D MERGE MR imaging data in the bifurcation region are shown for both baseline and follow-up scans (a,c). The vessel wall thickness maps superimposed on axial views are also shown (b,d). The axial images from the baseline and follow-up scans were meticulously chosen to represent identical anatomical regions; yet, the slices could not be fully aligned. The middle and right panels of Figure 7, illustrate extracted centerlines, bifurcation coordinate systems, and vessel wall thickness maps on luminal surfaces in baseline and follow-up scans for the right (e) and left (f) carotid arteries, respectively. We note that only CCA and ICA branches are considered. From the axial images shown, we see that image qualities in the bifurcation region for the baseline and follow-up scans are comparable; however, automated segmentation results reveal that the AI framework is not able to fully localize the distal CCA of the right carotid in the follow-up scan. This is due to signal-to-noise ratio (SNR) degradation occurring as one moves away from the bifurcation region, given that the carotid coils are conventionally centered around the carotid bulb.

Figure 8 compares the 3D vessel wall thickness measurements on luminal surfaces of the left and right carotid arteries between the baseline and follow-up scans. The luminal surfaces were partitioned into rectangular patches using bifurcation coordinate systems (see Figure 7e,f), subsequently unwrapped and flattened to generate regular 2D grids. Vertical red lines separate 2D grids associated with CCA and ICA branches. Each thickness value on the flattened grid is computed by averaging thickness measurements within the corresponding patch on the luminal surface. Using this approach, we can represent the thickness measurement results in a standardized structure, enabling quantitative and localized comparisons. This is achieved by computing the difference between flattened grids corresponding to baseline and follow-up scans, as demonstrated in Figure 8.

From Figure 8, it is evident that the wall thickness of both left and right carotid arteries predominantly falls within the range of 1 to 1.5 mm, with the exception of flow dividers in ICA branches. The latter can be readily identified on flattened thickness maps as dark blue spots. This observation implies a normal vessel wall condition for the subject under study, aligning with expectations for a healthy volunteer. The difference maps in Figure 8 demonstrate a close correspondence in thickness measurements between the baseline and follow-up scans. The small differences observed indicate a high reproducibility of the Laplacian method. Notably, the most significant differences manifest in the distal part of CCA branches, where the discrepancy may be attributed to inconsistent segmentation of the outer wall, potentially arising from the SNR degradation, as discussed previously.

Figure 9 compares the average 3D vessel wall thickness measurement, luminal radius, and the normalized wall volume index of the left and right carotid arteries along their respective centerlines between the baseline and follow-up scans. The solid-line curves correspond to the baseline, while the dash-line curves represent the follow-up scan. Carotid artery branches are color-coded, with orange indicating the CCA, magenta indicating the ICA, and cyan indicating the bifurcation region. From Figure 9, we see a close alignment between the 3D thickness measurements obtained from the baseline and follow-up scans, indicating a high level of reproducibility in 3D thickness measurements. Additionally, the reproducibility of the 3D thickness measurement, obtained using the Laplacian method, remains consistent with other morphological features, particularly the normalized wall volume index. The average vessel wall thickness for the subject under study falls within the range of 1 to 1.5 mm, while the normalized wall volume index ranges from 0.4 to 0.6, suggesting a normal vessel wall condition. In line with observations from Figure 7 and Figure 8, the most significant deviation between baseline and follow-up measurements is observed in the distal part of the CCA branch.

## 5. Discussion

In this paper, we advocated for the recognition of vessel wall thickness measurement as inherently a 3D challenge and proposed the adoption of the Laplacian method to address this challenge in medical imaging applications. We argued that this method is capable of overcoming limitations associated with existing 2D-centric approaches. Specifically, the Laplacian method is versatile in its application to both 2D and 3D scenarios. It captures variations in vessel wall thickness across all dimensions, without reliance on the extraction of centerlines and 2D cross-sectional slices. This method provides localized wall thickness measurements, enabling precise 3D localization of regions manifesting increased wall thickness. Furthermore, it can be implemented efficiently by leveraging convolutions available in deep learning platforms and harnessing GPU resources. We also demonstrated the accuracy and reproducibility of the proposed method through multiple experiments involving digital phantoms and actual medical imaging data.

Throughout this paper, in both theoretical and experimental settings, the thickness measurement using the Laplacian method is explained in scenarios characterized by isotropic pixel/voxel dimensions. Generalization of this method to accommodate non-isotropic pixel/voxel dimensions is straightforward and can be achieved by incorporating scale factors corresponding to distinct spatial dimensions. An alternative solution is to resample the original object ensuring that the resultant pixels/voxels have isotropic dimensions, and then utilizing the implementation explained in this paper.

In the course of experiments involving digital phantoms, it was observed that increasing the spatial resolution leads to improved accuracy of thickness measurements utilizing the Laplacian method. In practical scenarios, increasing the spatial resolution of the binary mask of an object through upsampling can yield more precise thickness measurements. However, this also results in a heavier computational load associated with the Laplacian method. This effect is particularly noticeable in the case of 3D objects, where an upsampling by a factor of *n* translates into a volumetric increase in the number of voxels by $n^{3}$. The computational demand primarily arises from the number of pixels/voxels for which the thickness measurement is required. To address the computational burden in higher spatial resolution scenarios, one strategy involves measuring the thickness within a subset of pixels/voxels and subsequently extrapolating the entire thickness map. A more rigorous approach entails dynamically tracking the streamlines and storing information about voxels that share the same streamline, as they inherently have identical thickness measurements.

As previously mentioned, thickness describes the distance separating two boundaries of an object or material. When the boundaries of an object lack clear delineation, utilizing the Laplacian method for thickness measurements may yield inaccurate results. Therefore, particular attention should be paid towards regions where the boundaries of an object are not well-defined. In the context of measuring vessel wall thickness, this scenario may arise when two vessels appear to be in close proximity, leading to potential inaccuracies. For instance, the internal and external carotid arteries in the carotid bifurcation region often exhibit close spatial proximity, and the outer wall boundary in the flow divider section may remain invisible in the imaging data due to the inherent spatial resolution limitations of the imaging technique. In such instances, it is essential to correct the segmentation masks of the vessels to ensure an accurate separation of the outer wall boundaries of distinct vessels.

## 6. Conclusions

In this paper, we studied the vessel wall thickness measurement using the Laplacian method and demonstrated its efficient implementation using convolutions. Additionally, we showcased the application of this technique in 2D and 3D scenarios by conducting multiple experiments and investigating its accuracy, reproducibility, and localization capabilities. Overall, the method proposed in this research offers a promising avenue for accurate vessel wall thickness measurement, delivering heightened localization and comprehensive assessments in both 2D and 3D scenarios. By addressing the limitations inherent in existing techniques, this method holds the potential to advance the field of cardiovascular imaging, contributing to more precise risk evaluation and targeted interventions.

## Figures and Tables

**Table 1:** Morphological measurements form the histology images.

ROI #	ROI Type	ROI Area (mm^2^)	Normalized Index	Wall Thickness (mm) (Mean ± Std)	Dist. to Lumen (mm) (Mean ± Std)
Sample #1					
1	Lumen	5.64	0.14	-	-
2	Outer Wall	34.30	0.86	2.24 ± 0.31	1.29 ± 0.66
3	Calcification	3.80	0.09	1.93 ± 0.09	1.44 ± 0.23
4	Hemorrhage	9.05	0.22	2.30 ± 0.36	1.40 ± 0.41
5	Lipid Pool	0.85	0.02	2.42 ± 0.06	1.84 ± 0.17
6	Lipid Pool	3.74	0.09	2.49 ± 0.16	0.52 ± 0.29
7	Lipid Pool	4.69	0.12	2.11 ± 0.20	1.02 ± 0.56
Sample #2					
1	Lumen	19.93	0.29	-	-
2	Outer Wall	49.77	0.71	2.31 ± 1.02	1.32 ± 0.96
3	Calcification	1.63	0.02	2.35 ± 0.25	1.86 ± 0.36
4	Hemorrhage	2.24	0.03	1.86 ± 0.23	1.15 ± 0.31
5	Loose Matrix	1.89	0.03	2.32 ± 0.28	1.41 ± 0.27
6	Hemorrhage	9.04	0.13	3.42 ± 0.30	1.61 ± 0.55
7	Lipid Pool	0.51	0.01	1.27 ± 0.08	0.81 ± 0.14
8	Lipid Pool	2.05	0.03	1.11 ± 0.11	0.63 ± 0.19

## Data Availability

The digital phantoms and implementation codes for the proposed thickness measurement method are available online at https://github.com/KazemHashemi/Vessel-Wall-3D-Thickness-Measurement.git (accessed on 1 August 2024).

## Supplementary Materials

The following supporting information can be downloaded at: https://www.mdpi.com/article/10.3390/jcdd11080249/s1, Section S1: Overview of the Laplacian Operator.

## Abbreviations

The following abbreviations are used in this manuscript:

2D	Two-dimensional
3D	Three-dimensional
MRI	Magnetic resonance imaging
DL	Deep learning
GPU	Graphics processing unit
ROI	Region of interest
CCA	Common carotid artery
ICA	Internal carotid artery
H&E	Hematoxylin and eosin
IRB	institutional review board
3T	Three Tesla
3D-MERGE	3D motion-sensitized rapid gradient echo
FOV	Field of view
AI	Artificial intelligence
VMTK	Vascular modeling toolkit
SNR	Signal-to-noise ratio

## Appendix A. Extension to 3D

In the case of three-dimensional volume data, the Laplacian operator can be approximated by employing the seven-point stencil finite difference formula [44]. The formulation of this approximation is provided below:

(A1) $$\nabla^{2}\phi_{0,0,0}=\phi_{-1,0,0}+\phi_{1,0,0}+\phi_{0,1,0}+\phi_{0,-1,0}+\phi_{0,0,1}+\phi_{0,0,-1}-6\phi_{0,0,0}.$$

This leads to updating the potential field through a convolutional operation when iteratively solving the 3D Laplace’s Equation (refer to line 4 of Algorithm 2):

(A2) $$\begin{array}{cc} \phi^{\left(i+1\right)} & =\frac{1}{6}\left[\begin{array}{c} \left[\begin{array}{ccc} 0 & 0 & 0\\ 0 & 1 & 0\\ 0 & 0 & 0 \end{array}\right]\left[\begin{array}{ccc} 0 & 1 & 0\\ 1 & 0 & 1\\ 0 & 1 & 0 \end{array}\right]\left[\begin{array}{ccc} 0 & 0 & 0\\ 0 & 1 & 0\\ 0 & 0 & 0 \end{array}\right] \end{array}\right]⊛\phi^{\left(i\right)}=K_{7-\mathrm{pt}}⊛\phi^{\left(i\right)} \end{array}$$

Furthermore, the computation of the gradient of the potential field in three-dimensional space can be achieved through the utilization of the Sobel–Feldman operator, which is implemented using convolution operations. The process can be outlined as follows:

(A3a) $$\phi_{x}=\frac{1}{32}\left[\begin{array}{c} \left[\begin{array}{ccc} 1 & 0 & -1\\ 2 & 0 & -2\\ 1 & 0 & -1 \end{array}\right]\left[\begin{array}{ccc} 2 & 0 & -2\\ 4 & 0 & -4\\ 2 & 0 & -2 \end{array}\right]\left[\begin{array}{ccc} 1 & 0 & -1\\ 2 & 0 & -2\\ 1 & 0 & -1 \end{array}\right] \end{array}\right]⊛\phi =G_{x}⊛\phi$$

(A3b) $$\phi_{y}=\frac{1}{32}\left[\begin{array}{c} \left[\begin{array}{ccc} 1 & 2 & 1\\ 0 & 0 & 0\\ -1 & -2 & -1 \end{array}\right]\left[\begin{array}{ccc} 2 & 4 & 2\\ 0 & 0 & 0\\ -2 & -4 & -2 \end{array}\right]\left[\begin{array}{ccc} 1 & 2 & 1\\ 0 & 0 & 0\\ -1 & -2 & -1 \end{array}\right] \end{array}\right]⊛\phi =G_{y}⊛\phi$$

(A3c) $$\phi_{z}=\frac{1}{32}\left[\begin{array}{c} \left[\begin{array}{ccc} 1 & 2 & 1\\ 2 & 4 & 2\\ 1 & 2 & 1 \end{array}\right]\left[\begin{array}{ccc} 0 & 0 & 0\\ 0 & 0 & 0\\ 0 & 0 & 0 \end{array}\right]\left[\begin{array}{ccc} -1 & -2 & -1\\ -2 & -4 & -2\\ -1 & -2 & -1 \end{array}\right] \end{array}\right]⊛\phi =G_{z}⊛\phi$$

## Appendix B. More Robust Kernels

The convolution operators employed in Equations (5) and (A2) utilize a minimal number of spatial points when computing the potential field’s Laplacian. However, a more stable and isotropic approach to implementing the Laplacian necessitates incorporation of additional spatial points. In the context of 2D space, this can be accomplished by employing the nine-point stencil finite difference formula, as presented in [30], yielding the following formulation:

(A4) $$K_{9-\mathrm{pt}}=\frac{1}{12}\left[\begin{array}{ccc} 1 & 2 & 1\\ 2 & 0 & 2\\ 1 & 2 & 1 \end{array}\right]$$

For 3D scenarios, more robust and isotropic implementation of the Laplacian operation can be attained through the utilization of the 19- or 27-point stencil finite difference formula [44], leading to the derivation of the following kernels:

(A5a) $$\begin{array}{cc} K_{19-\mathrm{pt}} & =\frac{1}{24}\left[\begin{array}{c} \left[\begin{array}{ccc} 0 & 1 & 0\\ 1 & 2 & 1\\ 0 & 1 & 0 \end{array}\right]\left[\begin{array}{ccc} 1 & 2 & 1\\ 2 & 0 & 2\\ 1 & 2 & 1 \end{array}\right]\left[\begin{array}{ccc} 0 & 1 & 0\\ 1 & 2 & 1\\ 0 & 1 & 0 \end{array}\right] \end{array}\right] \end{array}$$

(A5b) $$\begin{array}{cc} K_{27-\mathrm{pt}} & =\frac{1}{88}\left[\begin{array}{c} \left[\begin{array}{ccc} 2 & 3 & 2\\ 3 & 6 & 3\\ 2 & 3 & 2 \end{array}\right]\left[\begin{array}{ccc} 3 & 6 & 3\\ 6 & 0 & 6\\ 3 & 6 & 3 \end{array}\right]\left[\begin{array}{ccc} 2 & 3 & 2\\ 3 & 6 & 3\\ 2 & 3 & 2 \end{array}\right] \end{array}\right] \end{array}$$

The gradient kernels described in Equations (7) and (A3) exhibit two distinct types of filtering operations. Firstly, there is the central difference operation in the gradient direction, characterized by filter weights of [1, 0, −1]/2. Secondly, there are the smoothing (averaging) operations executed perpendicular to the gradient direction with filter weights of [1, 2, 1]/4. To enhance the rotational symmetry and numerical consistency of the gradient kernels, alternative smoothing operations can be employed. In [31], an optimization process is demonstrated to improve the gradient kernels. This optimization results in smoothing weights of [47, 162, 47]/256.
