# Supplementary material for: Efficient and Accurate 3D Thickness Measurement in Vessel Wall Imaging: Overcoming Limitations of 2D Approaches Using the Laplacian Method
Source: J Cardiovasc Dev Dis. 2024 Aug 15;11(8):249. doi: 10.3390/jcdd11080249 (PMC11354343; doi:10.3390/jcdd11080249)
Supplement: Supplementary file 1 [file jcdd-11-00249-s001.zip › jcdd-3090679-supplementary.pdf]

Supporting Information:
Efficient and Accurate 3D Thickness Measurement in Vessel Wall Imaging: Overcoming Limitations of 2D Approaches Using the Laplacian Method

SeyyedKazem HashemizadehKolowri [1,*,†], Ebru Yaman Akcicek [1], Halit Akcicek [1], Xiaodong Ma[1], Marina S. Ferguson[2], Niranjan Balu [2], Thomas S. Hatsukami [3], and Chun Yuan[1,2]

1 Department of Radiology and Imaging Sciences, University of Utah, Salt Lake City, UT 84108, USA; ebru.akcicek@utah.edu (E.Y.A.); halit.akcicek@utah.edu (H.A.); xiaodong.ma@hsc.utah.edu (X.M.); chun.yuan@hsc.utah.edu (C.Y.)

2 Department of Radiology, University of Washington, Seattle, WA 98195, USA; msf2@uw.edu (M.S.F.); ninja@uw.edu (N.B.)

3 Department of Surgery, Division of Vascular Surgery, University of Washington, Seattle, WA 98195, USA; tomhat@uw.edu

* Correspondence: s.hashemizadehkolowri@utah.edu

† Current address: The Utah Center for Advanced Imaging Research (UCAIR), 729 Arapeen Dr., Salt Lake City, UT 84108, USA.

S1 Overview of the Laplacian Operator

S1.1 Introduction

The Laplacian operator is an essential tool in image analysis and mathematical modeling. It is widely used for tasks such as edge detection in images and solving partial differential equations (PDEs). This supporting section provides an introduction to the Laplacian operator, covering its definition, discrete version, and applications with an emphasis on potential fields, edge detection, and PDEs.

S1.2 Second-Order Derivatives and the Laplacian Operator

To understand the Laplacian operator, it's useful to first consider second-order derivatives. The second-order derivative of a function measures how the rate of change of the function itself is changing. For a function $f(x, y)$, these second-order derivatives are:

- $\frac{\partial^2 f(x,y)}{\partial x^2}$: The rate of change of the gradient in the x-direction.
- $\frac{\partial^2 f(x,y)}{\partial y^2}$: The rate of change of the gradient in the y-direction.

The Laplacian operator combines these second-order partial derivatives:

$$\nabla^2 f(x, y) = \frac{\partial^2 f(x, y)}{\partial x^2} + \frac{\partial^2 f(x, y)}{\partial y^2}$$

In the context of a potential field (such as gravitational or electrostatic potential), the Laplacian of a potential function $\phi(x, y)$ gives insight into the behavior of the field:

- **Positive Laplacian**: Indicates a local minimum of the potential function; the potential is relatively lower compared to its surroundings (The gradients converge at the positive Laplacian).
- **Negative Laplacian**: Indicates a local maximum; the potential is relatively higher compared to its surroundings (The gradients diverge at the negative Laplacian).
- **Zero Laplacian**: Suggests that the potential function is in a steady-state configuration where the average value around any point is constant (The gradients are parallel at the zero Laplacian).

In physical terms, for a potential field, a zero Laplacian signifies that the potential is harmonically balanced in that region.

S1.3 Discrete Version of the Laplacian Operator

In digital image processing, functions are represented as discrete pixel values. The discrete version of the Laplacian operator approximates the continuous operator using these pixel values. For a grayscale image with pixel values $f(i, j)$, where i and j are pixel coordinates, the discrete Laplacian is approximated by:

$$\nabla^2 f(i, j) \approx f(i-1, j) + f(i+1, j) + f(i, j-1) + f(i, j+1) - 4f(i, j)$$

This formula calculates the difference between the sum of the values in the neighboring pixels and four times the value of the central pixel. It highlights areas where the intensity changes significantly, which is useful for detecting edges.

S1.4 Laplacian Operator Applications

Edge Detection: The Laplacian operator is particularly effective for edge detection in images. Edges represent areas where there is a rapid change in pixel intensity. When the Laplacian is applied to an image, it highlights regions where this rapid change occurs. Specifically:

- **Positive Values**: Often correspond to the edges of bright objects on a darker background.
- **Negative Values**: Typically correspond to the edges of dark objects on a brighter background.

In practice, the Laplacian is often combined with Gaussian smoothing (as in the Laplacian of Gaussian, or LoG) to reduce noise and improve edge detection. The Gaussian filter smooths the image to minimize noise, while the Laplacian detects edges by emphasizing rapid intensity changes.

Solving Partial Differential Equations: The Laplacian operator is crucial in solving PDEs, such as Laplace's equation:

$$\nabla^2 \phi(x, y) = 0$$

Laplace's equation describes steady-state conditions where there is no change over time, such as the equilibrium temperature distribution in a solid. The Laplacian in this context helps to determine how a function behaves over a domain.

A zero Laplacian indicates that the function $\phi(x, y)$ is in a state of equilibrium or steady state. In physical terms, it means that there are no local sources or sinks of the quantity being modeled (e.g., heat or electrical potential). The concept of zero divergence in this context means that the average rate of change of the function is balanced, with no net accumulation or depletion at any point.

S1.5 Summary

The Laplacian operator is a versatile tool in both image analysis and mathematical modeling. Understanding its definition through second-order derivatives, its discrete approximation for image processing, and its applications in edge detection and PDEs provides a solid foundation for effectively utilizing this operator. This overview aims to enhance the comprehension of the Laplacian's role in various contexts, making its applications more accessible and meaningful.
